# Hsp90 Affecting Chromatin Remodeling Might Explain Transgenerational Epigenetic Inheritance in *Drosophila*

**DOI:** 10.2174/138920208786241207

**Published:** 2008-11

**Authors:** Douglas M Ruden, Xiangyi Lu

**Affiliations:** Wayne State University, Institute for Environmental Health Sciences, 2727 2^nd^ Ave, Room 4000, Detroit, MI 48201, USA

## Abstract

Transgenerational epigenetic inheritance, while poorly understood, is of great interest because it might help explain the increase in the incidence of diseases with an environmental contribution in humans, such as cancer, diabetes, and heart disease. Here, we review five *Drosophila* examples of transgenerational epigenetic inheritance and propose a unified mechanism that involves Polycomb Response Element/Trithorax Response Element (PRE/TRE) occupancy by either Polycomb Group (PcG) protein complexes or Trithorax group (TrxG) complexes. Among their other activities, PcG complexes cause histone 3 lysine 27 tri-methylation associated with repressed chromatin, whereas Trithorax group (TrxG) complexes induce histone 3 lysine 4 tri-methylation associated with actively transcribed chromatin. In this model, Hsp90 is an environmentally sensitive chromatin remodeling regulator that causes a switch in the chromatin from a permissive state to a non-permissive state for transcription. Consistent with this model, Hsp90 has recently been shown to be a chaperone for Tah1p (TPR-containing protein associated with Hsp90) and Pih1p (protein interacting with Hsp90), which connect to the chromatin remodelling factor Rvb1p (RuvB-like protein 1)/Rvb2p in yeast [1]. Also, Hsp90 is required for optimal activity of the histone H3 lysine-4 methyltransferase SMYD3 in mammals [2, 3]. Since PcG and TrxG complexes are involved in the post-translational modifications of histones, and since such modifications have been shown to be required to maintain imprinted marks, this unified mechanism might also help to explain transgenerational epigenetic inheritance in humans.

## INTRODUCTION

In general, epigenetic modifications are established during early development in association with the differentiation of the various cell types and are cleared between generations in order to reestablish the totipotency of the zygote [4]. Recent reports of “heritable germline epimutations” at a couple of tumor suppressor genes in humans have reignited the controversy over the transgenerational inheritance of epigenetic marks in higher organisms. There is now strong evidence that at a small number of loci the epigenetic marks are not completely cleared in yeast, plants, *Drosophila* and mice. This is referred to as transgenerational epigenetic inheritance (TEI) and there is much interest over the nature of the mark that is directly inherited.

The potential role that TEI plays in human health is important to understand because, according to Suter and colleagues, “*any genomic sequence* is potentially subject to this process, which can create the equivalent of a temporary loss-of-function mutation” (emphasis added) [5]. Reik and colleagues proposed that the resistance of Interstitial A particles (IAPs), which are a common family of transposable elements, to methylation reprogramming might provide a mechanism for TEI in the mouse [6]. There are several studies that show TEI of mouse genes that have IAP insertions, such as *Agouti*^variable yellow^ and *Axin*^fused^, [4, 7, 8] but examples of TEI of endogenous genes in mice have not yet been reported to our knowledge. 

Recently, TEI has been implicated in a few families at the human tumor suppressor genes MLH1 [9, 10] and MSH2 [11], in association with an increased risk of colorectal cancer. These and other mammalian studies have focused on DNA methylation, specifically 5-methyl cystosine (^5me^C) at CpG dinucleotide sequences (the ‘p’ stands for ‘phosphate’). However, *Schizosaccharomyces pombe* [12-15] and *Drosophila melanogaster *(reviewed here) also display the transgenerational transfer of non-genetic information *via *the gametes, and there is little or no DNA methylation in either organism. 

The existence of ^5me^C in the DNA of *Drosophila* remains contentious because its only cytosine methyl transferase homologue, MT2, has been shown to be an aspartic acid tRNA methyltransferase with no identified DNA methyltransferase activity [16]. Nevertheless, several laboratories have reported that *Drosophila* has ^5me^C at very low levels in the early embryo, leaving open the possibility of its relevance in some processes. [17-22]. However, since histone modifications are required for the inititation and maintenance of imprints in mammals, it is likely that DNA methylation is downstream of PcG and TrxG complexes that modify histones. 

There are several possible mechanisms that might explain TEI and related processes such as imprinting. Several laboratories have proposed that histone modification is the more ancient system for imprinting, whereas DNA methylation, which is a more stable mark, would have evolved later to maintain imprinting [23, 24]. The possible role of histone dynamics in TEI has been discussed in a recent review [25]. For example, the Histone H3 variant CENP-A is epigeneticcally inherited in human neo-centromeres [25]. Another possible mechanism for TEI is heritable RNA in sperm [26]. For example, mouse sperm contain microRNAs that are able to repress expression of the Kit gene, thereby causing the tips of mouse tails to be white [27]. However, neither of these proposed mechanisms would presumably be responsive to the environment, which is a key requirement if epigenetics plays a significant role in evolution [28-31].

Recently, in a large screen for proteins that genetically interact with Hsp90, two novel Hsp90 co-chaperones were identified, Tah1p (TPR-containing protein associated with Hsp90) and Pih1p (protein interacting with Hsp90), which connect to the chromatin remodelling factor Rvb1p (RuvB-like protein 1)/Rvb2p and provide a clear link from Hsp90 to mechanisms of epigenetic regulation [1]. Rvb1p/Rvb2p are involved in ATP-dependent chromatin remodeling during transcriptional activation. Also, Hsp90 is required for optimal activity of the SET-domain-containing histone H3 lysine-4 methyltransferase SMYD3 [2, 3]. The TrxG proteins Trithorax and Ash1 also have SET domains with H3 lysine-4 methyltransferase activity [32]. Based on these and other studies, we propose a new mechanism for TEI in *Drosophila* that involves a possible role of Hsp90 in regulating Polycomb Group (PcG) and Trithorax Group (TrxG) complexes *via *chromatin remodeling. First, we briefly review some of what is known about these complexes.

## POLYCOMB GROUP (PcG) AND TRITHORAX GROUP (TrxG) COMPLEXES IN *DROSOPHILA*

The first *Polycomb* (*Pc*) mutation was identified over 60 years ago by Pam Lewis, [33] the wife of the late Ed Lewis who was the co-winner of the 1995 Nobel Prize for Physiology and Medicine for his life-long work on the Bithorax Complex (BxC). Normal male fruit flies have a thick set of bristles called sex combs on their front pair of legs that they use for grasping females during copulation. In *Pc* mutant flies, there are also sex combs on the second and third pairs of legs, hence the name Polycomb. It was not until 1978 that Ed Lewis first described the cuticular morphology of lethal embryos homozygous for Polycomb mutant alleles, and suggested that the Pc^+^ gene product acts as a repressor of genes in the Bithorax gene complex [34].

**Fig. (1) Regulation of histone methylation by the PcG and TrxG complexes.**
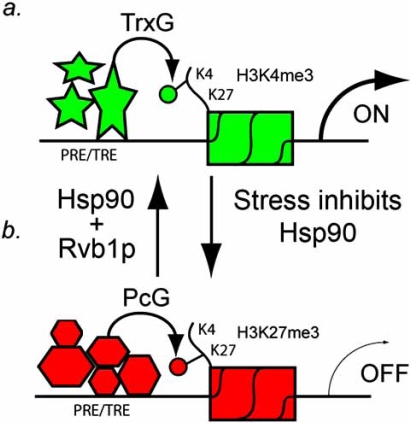
TrxG proteins (stars) form a complex on a PRE/TRE. Trithorax is a protein in the TrxG complex that tri-methylates Histone 3 lysine 4, which is an epigenetic mark that is associated with actively transcribed genes. In this model, Hsp90 is a chaperone for the chromatin-remodeling protein Rvb1p and/or TrxG proteins Trithorax and Ash1.Stress functionally inactivates Hsp90, thereby causing a replacement of the TrxG proteins at the PRE/TRE by PcG proteins (hexagons). Enhancer of zeste, E(z), is a protein in the PcG complex that tri-methylates Histone 3 lysine 27, which is an epigenetic mark that is associated with chromatin that is not permissive for transcription.

Currently, there are over a dozen members of the Pc Group (PcG) with similar phenotypes [35]. The PcG proteins and complexes are conserved from *Drosophila* to humans and are involved in the long-term maintenance of the repressed state of target genes during development (for review, see [32]). The precise mechanism by which PcG proteins maintain the repressed state of target genes is not known, but it is likely to involve the establishment of “repressive chromatin marks” on the histones, such as Histone 3 Lysine 27 tri-methylation (H3K27me3) by the PcG protein E(z) (Fig. **1B**) [36, 37]. 

The TrxG proteins and complexes are thought to counteract the repressive functions of the PcG proteins by inducing “active chromatin marks” on histones, such as Histone 3 Lysine 4 tri-methylation (H3K4me3) by Trithorax and Ash1, thereby allowing the long-term maintenance of the ‘activated’ (*i.e*., derepressed) state of the target genes (Fig. **1A**) [38, 39]. Both the PcG and TrxG complexes associate with PREs/TREs (Polycomb Response Elements/Trithorax Response Elements) which are several hundred base pairs long and have multiple transcription factor binding sites [40]. These sites are scattered throughout the genome at precise locations in *Drosophila*, but distinct locations of PREs/TREs in mammalian cells have not yet been identified [40]. Among their most well known functions, mutations in PcG genes derepress the BxC whereas mutations in TrxG genes inactivate expression of genes in this complex.

In Fig. (**1**), we propose that functional inactivation of Hsp90, by either mutation or environmental stress, can act to switch PRE/TRE occupancy from a TrxG protein bound state to a PcG protein bound state. Two novel Hsp90 co-chaperones were recently identified, Tah1p (TPR-containing protein associated with Hsp90) and Pih1p (protein interacting with Hsp90), which connect to the chromatin remodelling factor Rvb1p (RuvB-like protein 1)/Rvb2p [1]. Also, Hsp90 is required for optimal activity of the histone H3 lysine-4 methyltransferase SMYD3 [2, 3]. Since H3K4me3 is also catalyzed by the TrxG proteins Trithorax Ash1, [32] these findings suggest that stress-induced inactivation of Hsp90 might induce a switch from active chromatin to repressed chromatin that is no longer able to be transcribed. 

Work with Hsp90 heat-shock proteins by the Lindquist laboratory suggests that neutral genetic variation can accumulate in a population and can be freed under stressful environmental conditions [41, 42]. These authors argued that this could accelerate the pace of adaptive evolution under novel environmental conditions, and that Hsp90 functions as a “capacitor for morphological evolution” ^31 ^and a “capacitor for phenotypic evolution” [42]. The connection between Hsp90 and TrxG genes was first made by our laboratory which showed that inactivation of either Hsp90 or any of several TrxG proteins can affect TEI in *Drosophila *[43]. Because of this connection, we suggest that Hsp90 might be involved in the assembly or maintenance of the TrxG complex at the PRE/TRE *via *Rvb1p/Rvb2p-mediated chromatin remodeling (Fig. **1**). Since stress functionally inactivates Hsp90, this would provide an environmentally sensitive switch for conversion of chromatin from a permissive state (*i.e*., *via *TrxG) to a non-permissive state (*i.e*., *via *PcG). In the next sections, we discuss the possible role of Hsp90 and other chromatin-remodeling complexes in five examples of TEI in *Drosophila*.

## TEI SYSTEM 1: PCG AND TRXG COMPLEXES IN TRANSGENERATIONAL EPIGENETIC INHERITANCE IN *DROSOPHILA*

**Fig. (2) Metastable Epialleles in Drosophila and Humans.**
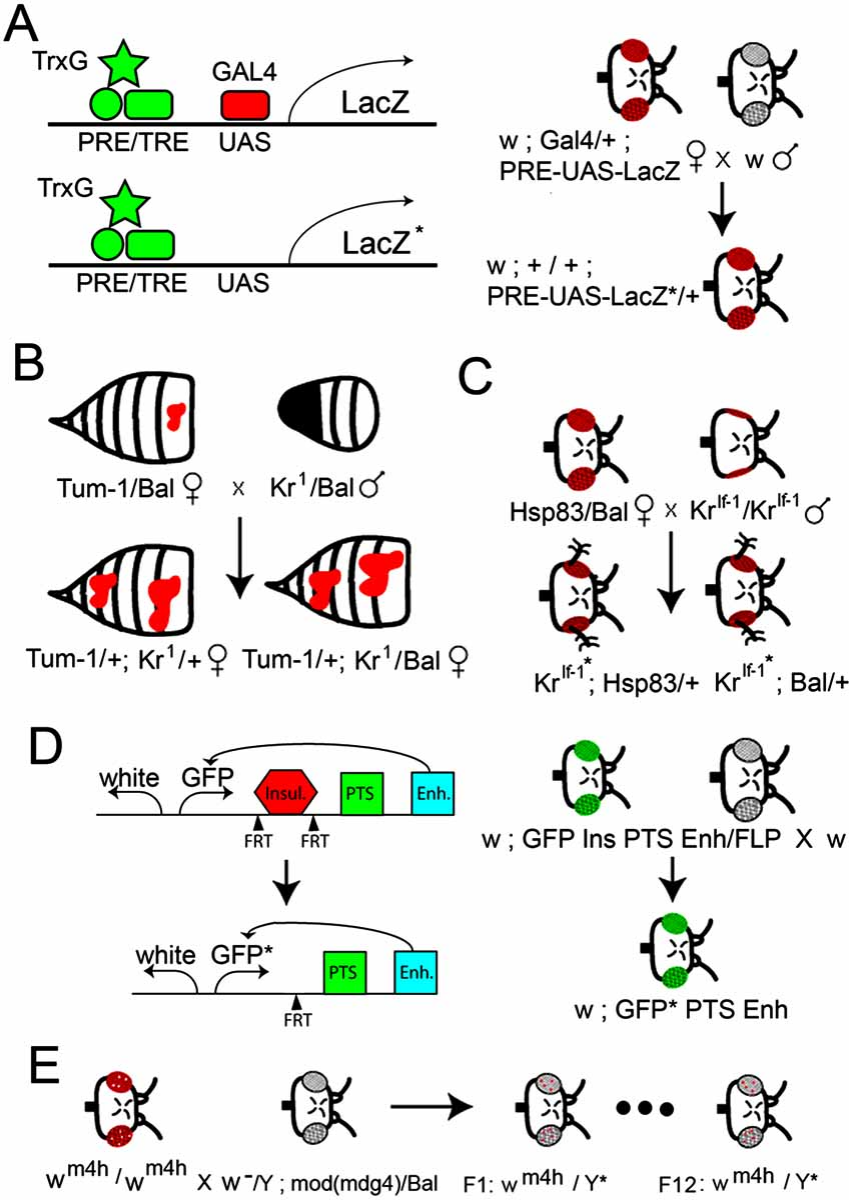
PRE/TRE-LacZ*, *Kr*^1^*, *Kr*^If-1^*, Enhancer-PTS-GFP*, and Y* are examples of transgenerational epigenetic inheritance because they can be transmitted through either or both the male and female germlines to subsequent generations. Left, The PRE/TRE-*Lac*Z* transgenerational epigenetic system in *Drosophila* is induced by maternal expression of GAL4, thereby activating expression of *LacZ* and replacing the PcG complex with a TrxG complex at the PRE/TRE site (PRE/TRE). The TrxG complex at the PRE-TRE does not require the inheritance of GAL4 in the affected progeny to maintain activation of *LacZ* [44, 45]. Right, The mini-*w*^+^ gene is downstream of *LacZ* and is also regulated by the PRE/TRE. The TrxG complex at the PRE/TRE also maintains activation of mini-*w*^+^ causing the eyes to be red in the progeny even in the absence of inherited Gal4 [44, 45].The *Kr*^1^* transgenerational epigenetic system in *Drosophila* is induced by maternal over-activation of JAK/STAT signaling, *via* *Hop*^Tum-1^, and paternal reduction of Krüppel (Kr), but does not require the inheritance of the non-functional Kr allele, *Kr*^1^, in the affected progeny [17]. The enhanced hematopoietic tumorigenic phenotype (large red blotches) induced by *Kr*^1^* is inherited in the next generation through the female germline. “Bal” is a “balancer chromosome” with a dominant marker that is used to maintain mutant stocks by preventing recombination [17].The *Kr*^If-1^* transgenerational epigenetic inheritance system is induced by maternal reduction of Hsp90 (Hsp83 in *Drosophila*) but does not require the inheritance of the non-functional Hsp83 allele in the affected progeny [43]. Once induced, the ELBO (ectopic large bristle out-growth) phenotype induced by *Kr*^If-1^* is transmitted through both male and female germlines through multiple generations [43].The Enhancer-PTS-GFP* transgenerational epigenetic system is induced by an Insulator (Ins.), such as a Su(Hw) binding site, but does not required the inheritance of the insulator in the affected progeny. Left, injection of the Enhancer-Insulator-PTS-GFP-white DNA construct can lead to either expression of GFP (shown) or *white* (w), but not both genes at the same time. PTS, Promoter Targeting Sequence, overcomes the Insulator and allows activation of either GFP (GFP*) or *white* (w*, not shown). GFP or *white* expression is maintained through multiple generations, even in the absence of the Insulator sequence. The Insulator which can be removed by recombining the FRT (FLPase-Recombinase Target) sites with FLP. The GFP* metastable epiallele is stable through at least 3 generations [55].The Y* (Y-imprinting) transgenerational epigenetic system is induced by a loss-of-function mutation in *mod(mdg4)*, but does not require the inheritance of the mod(mdg4) mutation in the affected progeny. The Y* allele shows an enhancement of variegation of the In(1)*w*^m4h^ chromosome for over 12 generations, which is as long as the experiment was carried out [62].

How might PcG and TrxG complexes be involved in TEI? The “genomic memory” systems mediated by PcG and TrxG proteins at PREs/TREs are especially attractive candidates for establishing and maintaining TEI because one only needs to invoke maintenance of the PcG and TrxG complexes in the germline in a similar manner to that already demonstrated during development. Indeed, Cavalli and Paro demonstrated almost a decade ago that the TrxG complex on a transgene carrying a PRE/TRE sequence is maintained in both the soma and the germline [44, 45]. Their experiments involved an artificial P-element transgene reporter system that contains a PRE/TRE followed by GAL4 binding sites regulating expression of *LacZ* (Fig. **2A**). On that reporter system, there was also a mini-* w*^+^ gene (adjacent to *LacZ* ) which was also regulated by the PRE/TRE (presumably *via *spreading of inactive chromatin over the entire region), thereby allowing easy visualization of the repressed or activated state by looking at the eye color (redness).

They showed that over expression of a strong transcriptional activator, GAL4, in the embryo (*via *heat shocking embryos with an *hsp70-GAL4* transgene) overcame the repressive/PcG state of the PRE/TRE and “switched” it to the active/TrxG state [44, 45]. The active/TrxG state was maintained throughout the larval mitotic divisions allowing efficient expression of both the *LacZ* and mini-* w*^+^ reporter genes. Remarkably, a significant percentage of mothers (but not fathers) transmitted the active/TrxG state to their progeny, even when the progeny did not inherit the *hsp70-GAL4* transgene (Fig. **2A**) [44, 45]. It is likely that GAL4-mediated transcriptional activation of the reporter genes requires the Rvb1p/Rvp2p chromatin remodeling proteins to disassemble the PcG complex because Rvb1p/Rvp2p, at least in yeast, are required for ATP-dependent chromatin-remodeling during general transcription [46-48] (Fig. **1**). Interestingly, these proteins have also recently been implicated in being required for epigenetic regulation of repressed chromatin near the telomeres in yeast [49]. 

Cavalli and Paro’s PRE/TRE-*LacZ* system meets the requirements of TEI because the active/TrxG state is inherited in more than one generation and no primary DNA sequence alterations are involved in setting the PRE/TRE switch [44, 45]. Furthermore, they showed that the PcG protein Polycomb is displaced from the PRE/TRE in the artificial construct when GAL4 is present (Fig. **2A**), and that mutations in the TrxG gene *trithorax* (*trx*) suppressed the transgenerational epigenetic inheritance [44, 45]. In 2003, Bantingnies and colleagues showed, using 3-dimensional FISH (fluorescent *in situ* hybridization) technology, that Polycomb-dependent chromosome interactions between the PRE in the transgene and a PRE in the endogenous Ubx gene are also stably meiotically inherited [50]. They showed that removing the “3-dimensional interactions” between the PREs by mutating the Ubx PRE caused stable epigenetic activation of the transgene for several generations, even when the Ubx PRE was restored in the F2 generation by backcrossing the PRE-mutant flies to wild type flies [50]. Interestingly, they also showed that elevated temperatures restores repression of the transgene [50]. which is consistant with our model of an Hsp90-inactivation mediated switch from a TrxG state to a PcG state at the PRE (Fig. **1**).

It would be interesting to determine whether Hsp90 inactivation affects TEI in their system, but this to our knowledge has not yet been tested. We speculate that mutations in Hsp90, or stress-inactivation of Hsp90, would suppress TEI in their system in a similar manner as they observe with *trx* mutations. Using their elegant polytene chromosome visualization system of PRE/TRE occupancy, and their FISH technology for characterizing 3-dimensional interactions, one could also determine whether Hsp90 is required for PRE/TRE occupancy and long-range chromatin interactions. Unfortunately, to our knowledge, such studies have not yet been conducted.

## TEI SYSTEM 2: GAMETIC EPIGENETIC INHERITANCE OF TUMORS IN *DROSOPHILA* 

Xing and colleagues [17] recently described a TEI system in *Drosophila* that is similar to that reported by Cavalli an Paro [44, 45]. Xing and colleagues identified suppressors and enhancers of *Hop*^Tum-1^, a dominant JAK kinase, which causes a hematopoietic tumorigenic phenotype (small dark blotches) in adult flies (Fig. **2B**) [51]. They showed that several of the enhancers of *Hop*^Tum-1^, including a loss-of-function allele of the Zn-finger transcription factor Krüppel, *Kr*^1^, demonstrate paternal inheritance [17]. For example, they found that *Hop*^Tum-1^/+ females mated to *Kr*^1^/+ males produced progeny (F1) with a significantly enhanced size and number of hematopoietic tumors, regardless of whether or not they inherited the *Kr*^1^ mutation (Fig. **2B**) [17].

Xing and colleagues attributed the *Kr*^1^ like phenotype in *Kr*^+^ offspring to DNA methylation at Kr target sites [17]. They demonstrated increased DNA methylation in a ftz promoter region that is regulated by Kr, and concluded that the aberrant ftz transcription and promoter methylation are both transgenerationally heritable. The role of *Hop*^Tum-1^, they argue, is that JAK over activation disrupts epigenetic reprogramming and allows inheritance of methylated Kr target sequences that influence tumorigenesis in future generations [17]. However, we argue, even if DNA methylation occurs in *Drosophila*, that DNA methylation would likely be downstream of PcG function as this is more likely to establish and maintain DNA methylation marks. We think that it is more likely that Rvb1p/Rvb2p or SMYD3/Trithorax inactivation by *Hop*^Tum-1 ^(which might reduce Hsp90 levels, see below) mediates the switch from active to inactive chromatin at the Kr PRE/TRE (Fig. **1**).

What are the implications of Xing and colleagues studies on the TEI of tumors? An analogous situation in humans would be an increased susceptibility to cancer in the offspring of cancer patients, regardless of whether they inherited any of their parent’s tumor susceptibility genes. Such a non-Mendelian inheritance system in epidemiological studies would generally be passed of as an “environmental” contribution. Feinberg and colleague are developing a more rigorous statistical system to account for TEI in cancer etiology, [52] but this is still in very early stages. 

## TEI SYSTEM 3: MULTI-GENERATIONAL EPIGENETIC INHERITANCE OF EYE OUTGROWTHS IN *DROSOPHILA *

In our TEI system, we used another allele of Krüppel, *Kr*^If-1^, which is caused by a repetitive sequence insertion in the *Kr* promoter [53] that induces ectopic over-expression of *Kr* mRNA and Kr protein in the eye imaginal disc [43, 54]. As did Li and colleagues with *Hop*^Tum-1^, we identified modifiers of the *Kr*^If-1^ “small eye” phenotype with unusual properties [43]. We determined that maternal reduction in Hsp90 (Hsp83 in *Drosophila*), or maternal reduction of any one of a number of TrxG genes, caused dramatic Ectopic Large Bristle Outgrowths (ELBOs) that often resembled proximal appendages protruding from the ventral regions of one or both eyes (Fig. **2C**). 

As Li and colleagues found with *Kr*^1^ in their TEI system, the maternal *Hsp83* or TrxG gene mutation was required to cause the ELBO phenotype, but the *Hsp83* or TrxG gene mutation was not required to be present in the affected progeny (Fig. **2C**). Moreover, we showed that ELBOs can be induced in an isogenic strain of *Drosophila* with the *Kr*^If-1^ mutation by feeding parents the specific and potent Hsp90 inhibitor geldanamycin, thus demonstrating that loss of Hsp90 activity and not something else in the genetic background had established a postulated *Kr*^If-1^ metastable epiallele [43]. 

An analogous system in humans would be a multigenerational increase in the severity of cancer in a manner that is independent on the accumulation of tumor causing genes. While speculative, such an accumulative TEI system in humans might explain the generational increase in environmentally sensitive diseases, such as diabetes, cardiovascular disease, autism, and cancer. Genetics alone cannot explain the dramatic increases in some of these diseases during that past few decades, but an epigenetics approach might help us finally reach a better understanding of the processes involved.

## TEI SYSTEM 4: EPIGENETIC INHERITANCE OF PROMOTER TARGETING SEQUENCE (PTS)-MEDIATED ACTIVATION OF ENHANCERS

A fourth example of TEI in Drosophila is promoter targeting sequence (PTS) mediated epigenetically heritable transcription memory that was identified by Lin and colleagues [55]. The PTS is an “anti-insulator” from the Abdominal-B locus of the BxC that is able to overcome an insulator sequence which normally blocks enhancer activation of promoter sequences [55]. Lin and colleagues determined using transgenic Drosophila strains that promoter targeting activity, once established, is stable for several generations (Fig. **2D**). In other words, a PTS-enhancer-insulator-reporter-containing transgene that has expression of the reporter gene will have stable expression of the reporter gene for several generations, whereas other transgene insertions will have stable repression of the reporter for several generations [55]. It would be interesting to determine whether Hsp90 is involved in PTS function, but this is currently not known.

## TEI SYSTEM 5: EPIGENETIC INHERITANCE OF Y-CHROMOSOME IMPRINTING IN *DROSOPHILA*

A fifth example of TEI in Drosophila is Y-chromosome imprinting (Fig. **2E**). Maggert and colleagues found that most P-element insertions on the heterochromatic Y chromosome of Drosophila showed differential expression of one or both genes according to the parental source of the chromosome [56]. They called this parent of origin effect Y-chromosome imprinting [56]. The Y-chromosome from Drosophila males suppresses positional effect variegation (PEV), in which the insertion or translocation of a gene near heterochromatin causes variegated expression [57]. For example, the In(1)wm4h rearrangement, which was isolated by Muller in 1930, have eyes with a strong white-mottled phenotype [58]. This rearrangement, which juxtaposes the white locus to centric X heterochromatin, has frequently been used for isolating PEV-modifying mutations [59, 60]. As with the PcG and TrxG proteins, many of the Su(var) and E(var) proteins are involved in post-translational modifications of the histones. For example, Su(var)3-9 is a histone 3 lysine 9 (H3K9) methyl transferase, and the H3K9me3 epigenetic mark is associated with regions of condensed chromatin that do not allow transcription of most genes [61]. 

Interestingly, mod(mdg4), also called E(var)3-3, affects Y-chromosome imprinting for several generations. Mutations in mod(mdg4) has been shown to reduce the effects of the Y chromosome on suppressing variegation by somehow imprinting the Y chromosome. Dorn and colleagues have shown that the Y-chromosome from mod(mdg4) males does not suppress variegation even in male offspring that do not inherit the mod(mdg4) mutation. This “paternal effect” phenotype is stable and lasts for at least 11 generations through the male germline, which is as long as the experiment was carried out. 

Interestingly, mod(mdg4), also called E(var)3-3, in addition to its enhancer of variegation activity, is also involved in regulation of homeotic gene complexes. Dorn and colleagues showed that mod(mdg4)homozygous mutant males showed significant transformation of the fifth into the fourth abdominal segment, which is also a characteristic of TrxG gene mutations [62]. Since Y chromosome imprinting resembles the KrIf-1* TEI system, it would be interesting to determine whether Hsp90 is involved.

## SIMILARITIES AMONG THE TRANSGENERATIONAL EPIGENETIC INHERITANCE SYSTEMS IN *DROSOPHILA*

In this review, we identify metastable epialleles of genes with an asterisk (*). We refer to the metastable epiallele of Cavalli and Paro [44, 45] as PRE/TRE-LacZ*, the metastable epiallele of Li and colleagues as Kr^1^* [17], and metastable epiallele of *Kr*^If-1^ as *Kr*^If-1^*, the metastable epiallele of GFP by the PTS as GFP*, and the metastable epigenetic modification of the Y-chromosome as Y* (Fig. **2**). The five metastable epialleles show similarities in their interactions with Hsp90 and/or TrxG proteins and other chromatin-remodeling complexes (Table **1**). First, the change in expression state at PRE/TRE-LacZ* is established by embryonic expression of GAL4 converting the PRE/TRE from the repressed/PcG state to the active/TrxG state (Fig. **2A**). The TEI phenotype is suppressed by mutations in *trx*. Second, the Kr^1^* metastable epiallele is induced by *Hop*^Tum-1^, but we speculate that it might also be induced by loss of Hsp90. It has been shown that activation of JAK, such as occurs in *Hop*^Tum-1^ cells, prevents Heat Shock Factor (HSF) from inducing expression of Hsp90 in heat-shocked cells [63-66] (Fig. **2B**). However, this has not yet been shown in *Drosophila.* Third, the *Kr*^If-1*^ epigenetic phenotype (ELBO) is established by reducing either Hsp90 or TrxG protein activity (Fig. **2C**). Fourth, the Y* imprinting Y-chromosome is enhanced by mutations in the TrxG genes trithorax, brahma, and verthandi (Fig. **2E**). 

What model might unify the five *Drosophila* TEI systems? We believe that all five systems might be partly explained if we propose that Hsp90 is required for TrxG complex formation by Rvb1p/Rvb2p chromatin remodeling proteins, or like the SET-domain containing protein SMYD3, [3] the SET domain proteins Trithorax or Ash1 require Hsp90 for optimal activity (Fig. **1**). This would allow an attractive evolutionary mechanism for induction of new heritable germline epimutations by the environment [28]. Hsp90 has been called a “capacitor for morphological evolution” by Lindquist and colleagues because reduction of Hsp90 activity releases previously masked abnormal morphologies, such as bent legs, rough eyes, and deformed wings [41]. What would make Hsp90 a unique member of the TrxG is that it is an environmentally responsive global regulator of transcription, rather than a constitutively active protein as are thought the other members of the TrxG.

In all five *Drosophila* TEI examples, we propose that TEI is established and maintained by a shift in the active/TrxG complex to the repressed/PcG complex at one or more PRE/TREs. It is possible that this switch involves long-range chromatin alterations, such as to “Polycomb Bodies” which have been proposed to be sites of repression of chromatin by PcG proteins in the nucleus [67, 68]. This hypothesis can be tested, in experiments that have not yet been done, by cytological examination of polytene chromsomes stained with anti-PcG antibodies, or by the 3-dimensional FISH technology used in Cavalli’s laboratory [50]. The model predicts that there will be more PcG proteins on the PRE/TRE at the wild-type locus compared with the metastable-epiallele locus, and that there will be more transcription from the wild-type gene in early embryos compared with the metastable-epiallele gene. Such experiments have not yet been completed, but they would help support or refute our hypothesis.

## CONCLUSIONS AND EXTENSIONS: TRANSGENERATIONAL EPIGENETIC INHERITANCE IN MICE AND MEN

How might the studies of five examples of TEI in *Drosophila* presented in this review (Fig. **2**) help us understand TEI in humans? As mentioned in the Introduction, there are currently only a couple examples of putative heritable epimutations in humans, [9-11] but the evidence that these are truly examples of TEI is slim [69]. It is possible that TEI in humans, should it exist, utilize similar mechanisms as *Drosophila*. Evidence already exists for repressor/PcG complexes being required for establishing DNA methylation during mammalian X inactivation and genomic imprinting [70-73]. It is attractive to invoke a similar mechanism for gametic epigenetic inheritance. In this regard it is worth noting that recent studies at the *Agouti viable yellow (A^vy^) *allele in mice, [74] suggest that the epigenetic mark is PcG-mediated [75]. 

While PRE/TRE sites have not been as well characterized in mice and humans as in *Drosophila*, this situation is rapidly improving with whole-genome chromatin mapping studies by ChIP (Chromatin Immuno-Precipitation) [76-79]. Using the ChIP approach, embryonic stem (ES) cells and other stem cells have been shown to have many genes containing “bivalent” chromatin marks consisting of both repressive (H3K27me3) and active (H3K4me3) marks [80, 81]. These “bivalent” states are hypothesized to “poise” relevant genes for future activation when the stem cells differentiate [80, 81]. Also, it has recently been shown that a human homolog of Kr (Klf4) is partly involved in the successful reprogramming of differentiated human somatic cells into totipotent stem cells [82]. This is especially interesting given that fact that metastable epialleles of Kr are involved in two of the *Drosophila* examples of TEI (Fig. **2B**,**C**). 

It will be interesting to determine whether switching of “bivalent” chromatin marks by alternative PcG and TrxG complexes at Klf4 and other stem cell genes is a mechanism for generating gametic epigenetic inheritance in mammals. Undoubtedly, future studies in *Drosophila* will help lead the way.

## Figures and Tables

**Table 1.** Comparisons of Five Transgenerational Epigenetic Systems in Drosophila

TEI System	Required Component	Removable Component	Role of Hsp90	Role of PcG/TrxG	Role of Suvar/Evar
PRE-UAS-lacZ*	PRE-UAS-lacZ	GAL4	ND	PcG for repression TrxG for activation	ND
*Kr*^1^*	Tum-1	*Kr*^1^	ND	ND	ND
*Kr*^If-1^*	*Kr*^If-1^	Hsp90, TrxG	Hsp90^-^ Inducer	TrxG^-^ inducer	Suvar^-^ suppresser Evar^-^ enhances
Enh-PTS-GFP*	Enh-PTS-GFP	Insulator	ND	ND	ND
Y*	Y chromosome	*mod(mdg4*)	ND	TrxG^-^ enhances	Evar^- ^enhances

The five TEI systems are described in Fig. (**2**). Required Component, are the gene, transgene, or chromosome required in all generations to see the epigenetic inheritance. Removable Component, the transgene or mutation that is required to induce the metastable epiallele but is not required for the subsequent maintenance of the epigenetic phenotype in subsequent generations. ND, not determined.
